# Protease-inhibiting, molecular modeling and antimicrobial activities of extracts and constituents from *Helichrysum foetidum* and *Helichrysum mechowianum* (compositae)

**DOI:** 10.1186/s13065-015-0108-1

**Published:** 2015-05-30

**Authors:** Fanny-Aimée Essombe Malolo, Achille Bissoue Nouga, Antoine Kakam, Katrin Franke, Lidwine Ngah, Otavio Flausino, Emmanuel Mpondo Mpondo, Fidele Ntie-Kang, Jean Claude Ndom, Vanderlan da Silva Bolzani, Ludger Wessjohann

**Affiliations:** Department of Pharmacy, University of Douala, Douala, P.O. Box 812, Cameroon; Department of Chemistry, University of Douala, Douala, P.O. Box 567 24157, Cameroon; Química, Departamento de Química Orgânica, Núcleo de Bioensaio, Biossíntese e Ecofisiologia de Produtos Naturais-NuBBE, Universidade Estadual Paulista (UNESP) Instituto de Rua Prof. Francisco Degni s/n, Araraquara, São Paulo 14.800-900 Brazil; Department of Bioorganic Chemistry, Leibniz Institute of Plant Biochemistry, Weinberg 3, D-06120 Halle (Saale), Germany; Chemical and Bioactivity Information Centre, Department of Chemistry, University of Buea, P. O. Box 63, Buea, Cameroon

**Keywords:** Compositae, *Helichrysum foetidum*, *Helichysum mechowianum*, ESI-MS, Protease inhibition assay, Antimicrobial

## Abstract

**Background:**

*Helichrysum* species are used extensively for stress-related ailments and as dressings for wounds normally encountered in circumcision rites, bruises, cuts and sores. It has been reported that *Helichysum* species are used to relief abdominal pain, heart burn, cough, cold, wounds, female sterility, menstrual pain.

**Results:**

From the extracts of *Helichrysum foetidum* (L.) Moench, six known compounds were isolated and identified. They were 7, 4′-dihydroxy-5-methoxy-flavanone **(1)**, 6′-methoxy-2′,4, 4′-trihydroxychalcone **(2),** 6′-methoxy-2′,4-dihydroxychalcone -4′-*O*-*β*-D-glucoside (**3**)**,** apigenin **(4),** apigenin-7-*O*-*β*-D-glucoside (**5**), kaur-16-en-18-oic acid **(6)** while two known compounds 3,5,7-trihydroxy-8-methoxyflavone (**12)**, 4,5-dicaffeoyl quinic acid (**13**) together with a mixture of phytosterol were isolated from the methanol extract of *Helichrysum mechowianum* Klatt. All the compounds were characterized by spectroscopic and mass spectrometric methods, and by comparison with literature data. Both extracts and all the isolates were screened for the protease inhibition, antibacterial and antifungal activities. In addition, the phytochemical profiles of both species were investigated by ESI-MS experiments.

**Conclusions:**

These results showed that the protease inhibition assay of *H. foetidum* could be mainly attributed to the constituents of flavonoids glycosides (**3, 5**) while the compound (**13**) from *H. mechowianum* contributes to the stomach protecting effects. In addition, among the antibacterial and antifungal activities of all the isolates, compound (**6**) was found to possess a potent inhibitor effect against the tested microorganisms. The heterogeneity of the genus is also reflected in its phytochemical diversity. The differential bioactivities and determined constituents support the traditional use of the species. Molecular modelling was carried out by computing selected descriptors related to drug absorption, distribution, metabolism, excretion and toxicity (ADMET).

**Graphical abstract:**
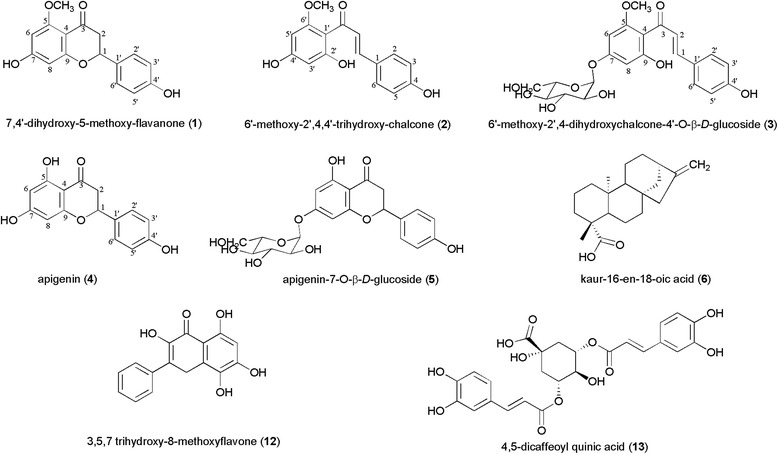
Compounds isolated from Helichrysum species (Compositae).

**Electronic supplementary material:**

The online version of this article (doi:10.1186/s13065-015-0108-1) contains supplementary material, which is available to authorized users.

## Background

The genus *Helichrysum* (Compositae) consists of more than 600 species with a major center of distribution in South Africa [1]. Several *Helichrysum* species have been used in folk medicine of different countries for the protection of post-harvest food [2]. Moreover, *Helichrysum* species are used extensively for stress-related ailments and as dressings for wounds normally encountered in circumcision rites, bruises, cuts and sores [3]. It has been also reported that *Helichysum* species are used to relief abdominal pain, heart burn, cough, cold, wounds, female sterility, menstrual pain [4] and to treat some diseases such as gastric [5–7], gastroduodenal, gastric ulcers and gastritis [8], stomach damage [9, 10], acute hepatitis, fever, or oedema [11], diuretic, inflammatory, allergic [12, 13]. In addition, some of these species have been reported to possess antimicrobially active compounds [14–16].

Chemical studies on *Helichrysum* species have been carried out by many investigators and the presence of flavonoids, phloroglucinols, α-pyrones, coumarins and terpenoid compounds has been reported [17–25]. *H. foetidum* has been assessed to treat influenza, infected wounds, herpes, eye problems, menstrual pains and to induce trance and possess antifungal properties [2, 26]. *H. mechowianum* is used for the treatment of stomach damage, cephalgy [9, 27] and possesses ulcerogenic activity [28, 29]. In continuation of these studies, **w**e extended our search for biologically active compounds from *Helichrysum* species [17, 18] to the protease-inhibiting activity of extracts and isolated compounds from *Helichrysum foetidum* and *Helichrysum mechowianum* using a fluorescence resonance energy transfer (FRET) protease pepsin inhibition assay as pharmacological model for anti-ulcer compounds [30]. Beside excessive stomach acid and *Helicobacter pylori*, pepsin is one of the major factors in the pathophysiology of peptic ulcer disease and reflux oesophagitis. In addition, the antibacterial and antifungal effects of both species against *Bacillus subtilis* and the yeast *Cladosporium cucumerinum* were evaluated respectively.

The chemical profile of methanol extracts of *H. mechowianum* and *H. foetidum* was investigated. To our knowledge, this is the first report about constituents of *H. mechowianum*. The compounds identified have been reported previously from other *Helichrysum* species in different compositions.

In order to assess the drug-likeness profiles of the isolated metabolites, low energy computer models were generated and a number of ADMET-related descriptors calculated, with the view of drug metabolism and pharmacokinetics (DMPK) evaluation.

## Results and discussion

### Biological tests

**Fig. 1:**
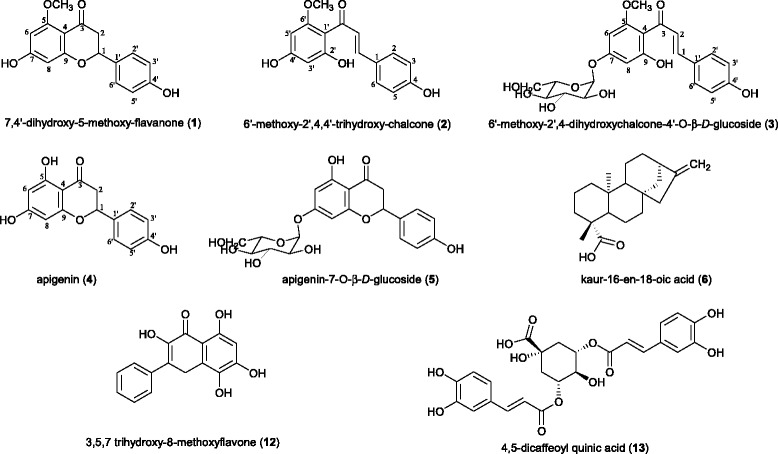
compounds 1–6 isolated from leaves and flowers of *H. foetidum* and *compounds* 12*–*13 from leaves of *H. Mechowianum*

The methanol leaf extracts of *Helichrysum foetidum* and *Helichrysum mechowianum* showed significant activity in the pepsin protease FRET assay while no activity was detected against the serine protease subtilisin (Table 1). The extract of *H. foetidum* exhibited the higher pepsin protease inhibition (37.4 and 35.6 % inhibition at 50 and 25 μg/ml) (Table 1). Therefore also the previously isolated constituents **1–6** (Fig. 1) from *H. Foetidum* and **12–13** from *H. mechowianum* were tested. The best results at a concentration of 50 μg/ml were obtained with apigenin-7-*O*-*β*-D-glucoside (**5**) and 6′-methoxy-2′,4-dihydroxychalcone-4′-*O*-*β*-D-glucoside (**3**) with a moderate inhibition activity of 46.3 and 37.4 % respectively (Table 1) while 3,5,7-trihydroxy-8-methoxyflavone (**12)**, 4,5-dicaffeoyl quinic acid (**13**) showed weak activity. These results suggest that the inhibition activity on the aspartate protease observed with *H. foetidum* extract could be mainly attributed to the glycosidic compounds (**3**) and (**5**). Contrarily, in the inhibition assay with the serine protease subtilisin, neither the crude extracts, nor the isolated substances of both species show significant activity (Table 1). We can conclude that the substances present in the crude extract of *H. foetidum* are selective for aspartate proteases. Observed negative results may be due to the auto-fluorescence debris of subtilisin cleavage of these compounds resulting in fluorescent residues or the absence of bioaffinity interactions between the substances present in the crude extract of *H. foetidum* with the serine protease subtilisin [31]. The observed protease inhibiting activity may have mucosal protective effects and therefore may help to reduce peptic ulceration. From the Black birch fungus (*Inonotus obliquus*)*,* which is used in folk medicine in Russia for the treatment of gastrointestinal tract disorders, also the flavonoidal fraction was shown to possess antiulcerous activity [31].

**Table 1:** Activity (% inhibition) of *Helichrysum* crude extracts and major isolated compounds (1–6) in protease inhibition assays using pepsin and subtilisin

	Inhibition of pepsin (%)					
Sample	Concentration (μg/ml)					
	50	25	10	1	0.1	0.01
*H. mechowianum*	22.8	18.8	12.0	0.3	10.9	5.4
*H. foetidum*	37.4	35.6	17.8	nd	12.6	7.1
1	nd	39.5	nd	−14.2	−1.5	0.4
2	nd	15.9	9.2	−1.4	nd	0.3
3	37.4	37.4	20.1	18.2	19.4	8.0
4	11.6	0.2	nd	0.2	7.8	1.8
5	46.3	37.2	15.1	18.6	−5.3	−2.9
6	25.0	−3.6	nd	−39.0	−1.2	−8.0
12	10.5	0.7	Nd	0.4	6.2	2.3
13	14.7	16.4	11.8	13.7	13.1	6.1
	Inhibition of subtilisin (%)					
*H. mechowianum*			−4.8	0.2	3.0	−0.1
*H. foetidum*			0.9	2.1	4.0	3,8
1			nd	nd	nd	nd
2			−11.6	−26.2	13.2	nd
3			9.7	4.2	6.0	3.0
4			11.5	7.8	6.8	nd
5			9.2	0.3	1.0	−4.8
6			−11.6	nd	−5.5	16.6
12			11.1	8.9	7.5	Nd
13			8.3	5.2	7.1	3.2

*nd* not detected

In addition, the crude extract of both species and all the isolated compounds were subjected to in vitro antimicrobial assay against the reference strains of bacteria *Bacillus subtilis* and yeast (*Cladosporium cucumerinu*).

It has been reported that extracts having MIC values below 8000 μg/ml possess some antimicrobial activity [32]. MIC values below 1000 μg/ml are considered noteworthy [33, 34]. Thus, the crude extract having activities of 1000 μg/ml or lower against all the pathogens studied demonstrated potential anti-infective properties. Compounds having antimicrobial activities less than 64 μg/ml are accepted as having notable antimicrobial activity [33] and those compounds exhibiting activity at concentrations below 10 μg/ml are considered “clinically significant” [32, 33]. According to this observation, the crude leaf and flower extracts of *H. foetidum* showed a significant and concentration dependent growth inhibition of *Bacillus subtilis* of of 85.4 % at a concentration of 1 mg/ml and of 21.8 % at a concentration of 0.1 mg/ml whereas the crude extract of *H. mechowianum* at concentrations of 1 mg/ml and 0.1 mg/ml causes a moderate growth inhibition of 36,2 and 29,8 % respectively (Table 2). Likewise, the crude leave and flower extracts of *H. Foetidum* also exhibit antifungal activity against *Cladosporium cucumerinu* shown by the development of inhibition zones on the bioautography plate. In contrast, extracts of *H. mechowianum* were slightly active against this fungus.

**Table 2:** Antimicrobial activity ((%)of *Helichrysum* crude extracts and isolated compounds 1–6, 12–13 from *H. Foetidum*

	Minimum inhibitory concentration ((%)			
Sample	Concentration (mg/ml)			
	*Cladosporium cucumerinum Bacillus subtilis*			
	1 0.1 1 0.1			
*H. mechowianum*	13.8	10.4	36.2	29.2
*H. foetidum*	71.3	17.7	85.4	21.8
1	68.5		76.3	
2	70.0		77.8	
3	66.7		78.2	
4	59.6		75.1	
5	56.1		81.6	
6	69.1		85.0	
12	NT		40.2	
13	NT		30.8	
AMP	74.5			
CIP			94.2	

*CIP* Ciprofloxacin antibacterial standard, *AMP* Amphotericin-B antifungal standard, *nd* not detected

Furthermore, all of the isolated compounds were subjected to in vitro antimicrobial assay. It was interesting to note that compounds **(1–6)** from *H. Foetidum* exhibited notable growth inhibition range of 85.0 to 75.0 % against *Bacillus subtilis* and a range of 70 to 56 % against the yeast *Cladosporium cucumerinu* at a concentration of 1 mg/ml whereas compounds **(12–13)** from *H. mechowianum* showed a moderate growth inhibition range of 40.2 to 30.8 % at 1 mg/ml against *Bacillus subtilis* (Table 2). Of all the isolated, compound (**6**) exhibited the highest sensitivity growth inhibition of *Bacillus subtilis* of 85.0 % at a concentration of 1 mg/ml and was found to be the most active component of the crude flower extract of *H. Foetidum* (Table 2).

The results of the work indicate that diterpenoid possess antimicrobial against the gram positive bacterium. This antibacterial activity of *H. foetidum* extract might be associated to the high content of kaurenoic acid (**6**). This justifies the use of these plants species in folk medicine and corroborated with the previous reports on the antibacterial activities for *Helichrysum* species [2, 35, 36]. Kaur-16-en-19-oic acid isolated from extract of the Asteraceae (*Senecio erechtitoides* and *Wedelia calendulaceae*) was previously shown to possess high inhibitory activity against several bacterial strains [37, 38].

### Chemical constituents

The main constituents of both species were characterized by detailed ESI-MS investigations. The combination of LC-MS, MS/MS and FTICR-HRMS allowed the detection of various components simultaneously. The MS experiments show, that *H. foetidum* and *H. mechowianum* possess different chemical compositions. The leaf extract of *H. foetidum* is dominated by the chalcones **2** and **3**, the flavonoids **4** and **5** and by diterpenoids [18] whereas main constituents of *H. mechowianum* are quinic acid derivatives with a less prominent bioactivity profile.

A more detailed ESI-MS investigation of the crude extract of *Helichrysum mechowianum* indicates (Additional file 1) the presence of quinic acid (**7**, ESI-FTICR-MS: [M - H]^−^, *m/z* 191.05578, calc. for C_7_H_11_O_6_^−^ 191.05556, ferulic acid (**8**, ESI-FTICR-MS: [M - H]^−^, *m/z* 193.05028, calc. for C_10_H_9_O_4_^−^ 193.05008, chlorogenic acid (**9**, ESI-FTICR-MS: [M - H]^−^, *m/z* 353.08751, calc. for C_16_H_17_O_9_^−^_,_ 353.08726, three isomers of dicaffeoyl quinic acid (**10**, ESI-FTICR-MS: [M - H]^−^, *m/z* 515.11949, calc. for C_25_H_23_O_12_^−^ 515.11895, and three isomers of methyl derivatives of **10** (**11**, ESI-FTICR-MS: [M - H]^−^, *m/z* 529.13539 calc. for C_26_H_25_O_12_^−^ 529.46950.

Compounds **7**–**11** were detected before in other *Helichrysum* species. Mono- and dicaffeoyl quinic acids are the main constituents of the Mediterranean herb *H. italicum* [38, 39] and are also present in the French *H. stoechas var. olonnense* [40]. Both are used as digestive. A similar compound composition is known for the Artichoke; *Cynara scolymus* L., which is used for its choleretic, lipid-lowering, hepatostimulating, and appetite-stimulating actions [41]. Extracts and constituents of artichoke were also shown to possess antibacterial and antifungal activities, however, Extracts and constituents of *H. mechowianum* showed least efficiency antifungal properties against the yeast *Cladosporium cucumerinum*. The observed quinic acid derivatives might be responsible for the stomach protecting effects of *H. mechowianum.*

Chromatographic separation of the partitioned extracts of *H. mechowianum* resulted in the isolation of a phytosterol mixture from the *n*-heptane fraction, 3,5,7-trihydroxy-8-methoxyflavone (**12**, ESI-FTICR-MS: [M + Na]^+^*m/z* 323.05297 calc. for C_16_H_12_O_6_Na 323.05261) from the ethyl acetate fraction and 4,5-dicaffeoyl quinic acid (**13**, ESI-FTICR-MS: [M - H]^−^, *m/z* 515.12168 calc. for C_25_H_23_O_12_^−^ 515.11950) from the water fraction. The relative composition of the phytosterol fraction was determined by GCMS as campesterol (2 %), stigmasterol (9.3 %), campest-7-en-3-ol (61.4 %), chondrillasterol (18.9 %), β-sitosterol (61.4 %) and an unidentified sterol (1.2 %). The compounds (**12**) [42] and (**13**) [43] were identified by comparison of spectral data with literature data. In addition, the position of the caffeoyl residues in compound (**13**) was determined by 2D NMR measurements. In particular, HMBC correlations from H-4 and H-5 of the quinic acid to C-9′, C-8′ and C-7′ of the caffeoyl residues indicates the substitution at position 4 and 5 (Table 3). Since this compound is reported to possess cytotoxic and apoptose inducing activity [44, 45], the anticancer activity against the prostate cancer cell line PC-3 was tested. However in concentrations of 50 nM or 50 μM no effect was observed with this cell line (Table 4).

**Table 3:** ^1^H and ^13^C NMR assignments for compounds (1–5, 12)

N^0^	1		2		3		4		5		12	
	^1^H (DMSO-d_6_)	^13^C (DMSO-d_6_)	^1^H (MeOD + CDCl_3_)	^13^C (MeOD + CDCl_3_)	^1^H (MeOD)	^13^C	^1^H (DMSO-d_6_)	^13^C (DMSO-d_6_)	^1^H (CD_3_OD)	^13^C (CD_3_OD)	^1^H (CDCl_3_)	^13^C (CDCl_3_)
1				128.0		128.0						
2	5.32 (1H, dd, 12.6, 2.9)	79 .0	7.49 (1H, d, 8.6)	131.0	7.51 (1H, d, 8.7)	131.0		164.4		163.0		136.5
3	2.51 (H3a)	42 .3	6.84 (1H, d, 8.6)	116.6	6.83 (1H, d, 8.7)	116.6	6.78 (1H, s)	106.5	6.58 (1H,s)	103.1	6 .70 (1H, s, OH)	144.9
	2.98 (H3b)											
4		196 .3		160.5		160.5		180.4		182.0		175.6
5	3.72 (OMe)	163.0	6.84 (1H, d, 8.6)	116.6	6.83 (1H, d, 8.7)	116.6	12.96 (1H, s, OH)	164.94		164.3	11 .49 (1H, s, OH)	156.6
6	6.04 (1H, d, 2.2)	104.6	7.49 (1H, d, 8.6)	131.0	7.51 (1H, d, 8.7)	131.0	6.19 (1H, d, 2.0)	104.8	6.83 (1H,d, 2.1 Hz)	99.9	6.33 (1H,s)	130.4
7	9 .60 (OH)	167.1					OH	160.1		157.0	6 .46 (1H, s, OH)	155.4
8	5.94 (1H, d, 2.2)	95.4					6.48 (1H, d, 2.0)	99.3	6.71 (1H,d, 2.1)	99.6	4.05 (3H, s, OMe)	148.0
9		162.8						160.7		161.1		155.4
10		101.6						109.3		105.4		130.7
1′		129.6		106.3		106.3		123.1		121.0		126.9
2′	7.28 (1H, d, 8.6)	127.6		165.9	7.51 (1H, d, 8.7)	165.9	7.93 (1H, d, 8.8)	129.3	7.83 (1H,d, 8.8)	128.7	8.24 (2H, d, 7.1)	128.8
3′	6.77 (1H, d, 8 .6)	115.0	5.99 (1H, d, 2.2)	92.2	6.31 (1H, d, 2.2)	92.2	6.93 (1H, d, 8.8)	117.0	6.92 (1H,d, 8.8)	116.0	7.60 (1H,m)	127.5
4′	9 .60 (1H, s, OH)	157.6		168.2		168.2	OH	162.6		161.5	7.49 (1H,m)	98.2
5′	6.77 (1H, d, 8 .6)	115.0	5.96 (1H, d, 2.2)	96.9	6.24 (1H, d, 2.2)	96.9	6.93 (1H, d, 8.8)	117.0	6.92 (1H,d, 8.8)	116.0	7.60 (1H,m)	127.5
6′	7.28 (1H, d, 8.6)	127.6		164.2	7.51 (1H, d, 8.7)	164.2	7.93 (1H, d, 8.8)	129.3	7.83 (1H,d, 8.8)	128.7	8.24 (2H, d, 7.1)	128.8
OMe-5	3.72	55.4										
OMe-6′			3.93	56.2	3.95	56.2						
OMe-8												61.9
			7.73 (1H, d, 16.0, Hα)	125.2 (CH-α)	7.72 (1H, s, Hα)	125.2						
			7.68 (1H, d, 16.0, Hβ)	143.5 (CH-β)	7.71 (1H, s, Hβ),	143.5						
				193.6 (C = O)		193.6 (C = O)						
1″					4.99 (1H, d, 7.5)	103.7			4.90 (1H,d) 7.6	105.1		
2″					4.24	71.0			3.50-3.25 (4H,m)	74.8		
3 ″’					3.61	77.2			3.50-3.25 (4H,m)	77.5		
4″					3.60	78.9			3.50-3.25 (4H,m)	71.8

**Table 4:** LC/MS data, deprotonated and protonated molecules (*m/z*) for peaks, including the retention times (Rt), MS/MS experiments of the constituents found in MeOH extract of *Helichrysum mechowianum* and *Helichrysum foetidum*

*Helichrysum mechiowianum*				*Helichrysum foetidum*			
Rt (min.)	HR-MS (*m/z*) from [M-H]^−^,	Molecular formula	Identified compounds by ESIMS	Rt (min.)	HR-MS (*m/z*) from [M-H]^−^, [M + H]^+^, [M + K]^+^ (%)	Molecular formula	Identified compoundsby ESIMS
9.1	191.055777[M-H]^−^	C_7_H_12_O_6_	quinic acid	16.1	455.0954180 [M + Na]^+^ 431.0992290 [M-H]^−^	C_21_H_20_O_10_	apigenin-7-*O*-*β*-glucoside
9.7	353.087674 [M-H]^−^	C_16_H_18_O_9_	chlorogenic acid	17.1	487.0992980 [M + K]^+^447.1293680 [M-H]^−^	C_22_H_24_O_10_	6′-methoxy-2′,4-dihydroxychalcone -4′-*O*-*β*-D-glucoside
10.2	193.050282[M-H]^−^	C_10_H_10_O_4_	ferulic acid	18.6	269.0449150 [M-H]^−^	C_15_H_10_O_5_	Apigenin
13.9/15.0/15.6	529.135391 [M-H]^−^	C_25_H_24_O_12_	Mixture of three dicaffeoyl quinic acid	19.5	287.0919010 [M-H]^−^	C_16_H_16_O_5_	6′-methoxy-2′,4, 4′-trihydroxychalcone
15.7/16.3/16.6	529.135391 [M-H]^−^	C_26_H_26_O_1_	Mixture of thtree dicaffeoyl quinic acid methyl ether	21.4	301.2166660 [M-H]^−^	C_20_H_30_O_2_	kaur-16-en-18-oic acid (5β,8α,9β,10α,13α)

### In silico pharmacokinetics assessment

Many bioactive compounds do not make it to clinical trials because of adverse pharmacokinetic properties. It therefore becomes imperative to access the pharmacokinetic profiles of potential drugs early enough in order to access their potential for further development. A summary of twenty two of the computed molecular descriptors used to assess the drug-likeness profiles of the isolated metabolites have been summarized in Table 2. These include the #stars or ‘drug-likeness’ parameter, the molecular weight (MW), the solvent accessible surface area (SASA), along with its hydrophobic component (FOSA) and hydrophilic component (FISA), the molecular volume, the number of hydrogen bond acceptors (HBA) and donors (HBD), the *n*-octanol/water partition coefficient (log P), the solubility parameter (log S), the predicted IC_50_ values for the blockage of the human-ether-a-go-go potassium ion (HERG K^+^) channels (logHERG), predicted permeability of Caco-2 cells, the blood–brain barrier partition coefficient (log BB), permeability of Madin-Darby canine kidney (MDCK) monolayers, skin permeability (log K_p_), the number of predicted primary metabolites (#metab), the binding affinity to human serum albumin (log K_HSA_), the percentage human oral absorption (PHOA), the number of violations of Lipinski’s ‘Rule of Five’ (Ro5) and Jorgensen’s ‘Rule of Three’ (Ro3) and the polar surface area (PSA). The range of values of each parameter for 95 % of known drugs have been given beneath Table 2. Five of these compounds (1, 2, 3, 5 and 12) showed #star = 0, which indicates that all the computed parameters fell within the recommended range for 95 % of known drugs. Meanwhile, compounds 4 and 6 showed only #star = 1. An overall ADME-compliance score, drug-likeness parameter (indicated by #stars), was used to assess the pharmacokinetic profiles of the isolated compounds. The #stars parameter indicates the number of property descriptors computed by QikProp [46], which fall outside the optimum range of values for 95 % of known drugs. The methods implemented were developed by Jorgensen *et al.* [47–49] (Table 5).

**Table 5:** Computed molecular descriptors for the assessment of the DMPK profiles of the major isolated metabolites and the recommended range for 95 % of known drugs

Metabolite	^*a*^#stars	^*b*^CNS	^*c*^MW (Da)	^*d*^SASA	^*e*^FOSA	^*f*^FISA	^*g*^volume	^*h*^HBD	^*i*^HBA	^*j*^log P	^*k*^log S
1	0	-1	286.3	506.7	157.9	145.1	880.6	2	5	1.9	-3.3
2	0	-2	286.3	548.9	110.0	181.5	920.6	2	4	2.3	-3.4
3	0	-2	270.2	491.3	0	200.4	827.7	2	4	1.7	-3.4
4	1	-2	448.4	734.5	249.3	263.0	1311.1	5	13	0.4	-3.1
5	0	-2	448.4	683.4	261.7	245.6	1264.2	5	14	-0.1	-3.0
6	1	-1	304.5	523.0	419.7	77.8	1007.6	1	2	4.8	-4.9
12	0	-2	284.3	504.1	26.4	215.2	856.2	3	4	1.5	-3.2
13	5	-2	516.5	839.4	183.4	^*^386.8	1495.9	^*^7	11	1.1	-4.5
Metabolite	^*l*^logHERG	^*m*^Caco-2	^*n*^log BB	^*o*^MDCK	^*p*^log K_p_	^*q*^#metab	^*r*^log K_HSA_	^*s*^PHOA	^*t*^Ro5	^*u*^Ro3	^*v*^PSA
1	-4.49	417.0	-0.9	192.2	-3.2	5	-0.06	84.7	0	0	83.4
2	-5.45	188.1	-1.7	81.3	-3.2	4	-0.04	80.9	0	0	93.5
3	-5.06	124.7	-1.4	52.1	-3.9	3	-0.01	74.4	0	0	101.0
4	^*^-6.01	31.8	-3.1	11.9	-4.2	7	-0.79	43.2	1	1	168.1
5	-5.17	46.5	-2.4	17.9	-4.6	8	-0.71	56.4	0	1	162.1
6	-0.99	459.4	-0.1	271.4	-2.8	2	0.79	^*^100	0	0	42.7
12	-4.97	90.3	-1.6	36.8	-4.2	5	-0.10	70.5	0	0	109.1
13	-4.99	^*^0.5	^*^-4.9	^*^0.2	-6.3	6	-0.63	0	3	1	235.6

^*^Property which falls outside the recommended range for 95 % of known drugs; ^*a*^Number of computed properties which fall outside the required range for 95 % of known drugs (recommended range 0 to 5); ^*b*^ Activity in the central nervous system in the scale −2 (inactive) to +2 (active); ^*c*^Molar weight (range for 95 % of drugs: 130–725 Da); ^*d*^The solvent accessible surface area (recommended range 300.0 to 1000.0 Å^2^); ^*e*^The hydrophobic component of the solvent accessible surface area (recommended range 0.0 to 750.0 Å^2^); ^*f*^The hydrophilic component of the solvent accessible surface area (recommended range 7.0 to 330.0 Å^2^); ^*g*^Total volume of molecule enclosed by solvent-accessible molecular surface, in Å^3^ (probe radius 1.4 Å) (range for 95 % of drugs: 500 to 2000 Å^3^); ^*h*^Number of hydrogen bonds donated by the molecule (range for 95 % of drugs: 0 to 6); ^*i*^Number of hydrogen bonds accepted by the molecule (range for 95 % of drugs: 2–20); ^*j*^Logarithm of partitioning coefficient between *n*-octanol and water phases (range for 95 % of drugs: −2 to 6.5); ^*k*^The predicted aqueous solubility, with S in mol/dm^3^ (range for 95 % of drugs: −6.5 to 0.5); ^*l*^Predicted IC_50_ value for blockage of HERG K^+^ channels (concern < −5); ^*m*^Predicted apparent Caco-2 cell membrane permeability in Boehringer–Ingelheim scale, in nm/s (range for 95 % of drugs: < 5 low, > 500 high); ^*n*^Logarithm of predicted blood/brain barrier partition coefficient (range for 95 % of drugs: −3.0 to 1.0); ^*o*^The predicted apparent MDCK permeability in nm/s (<25 poor, > 500 great); ^*p*^The predicted skin permeability (range for 95 % of drugs: −8.0 to −1.0); ^*q*^Number of likely metabolic reactions (range for 95 % of drugs: 1–8); ^*r*^Logarithm of predicted binding constant to human serum albumin (range for 95 % of drugs: −1.5 to 1.5); ^*s*^The predicted percentage human oral absorption (>80 % high, < 25 % poor); ^*t*^Number of violations of Lipinski’s ‘Rule of Five’ (Recommended maximum 4); ^*u*^Number of violations of Jorgensen’s ‘Rule of Three’ (Recommended maximum 3); ^*v*^Van der Waals surface area of polar nitrogen and oxygen atoms (range for 95 % of drugs: 7.0 to 200.0 Å^2^)

## Materials and methods

### General methods

Silica gel (Merck, 63–200 μm) and Sephadex LH-20 (Supelco) were used for column chromatography. Fractions were monitored by TLC using precoated silica gel plates 60 F_254_ (Merck). Spots were visualized by heating silica gel plates sprayed with vanillin-H_2_SO_4_ in MeOH. The ^1^H and ^13^C NMR spectra were recorded on a Varian Mercury 300 spectrometer at 300.22 and 75.50 MHz, respectively. ^1^H and 2D NMR spectra were recorded on a Varian VNMRS 600 system operating at a proton NMR frequency of 599.83 MHz equipped with a 5 mm inverse detection cryoprobe using standard CHEMPACK 4.1 pulse sequences (COSY, ROESY, 1DNOESY, HSQCAD, HMBCAD) implemented in Varian VNMRJ 2.2C spectrometer software. Chemical shifts were referenced to internal TMS (δ = 0 ppm, ^1^H) and CDCl_3_ (δ = 77.0 ppm, ^13^C) or CD_3_OD (δ = 49.0 ppm, ^13^C), respectively. The high resolution ESI mass spectra were obtained from a Bruker Apex III Fourier transform ion cyclotron resonance (FTICR) mass spectrometer (Bruker Daltonics, Billerica, USA) equipped with an Infinity™ cell, a 7.0 Tesla superconducting magnet (Bruker, Karlsruhe, Germany), an RF-only hexapole ion guide and an external APOLLO electrospray ion source (Agilent, off axis spray, voltages: endplate, -3.700 V; capillary, −4.200 V; capillary exit, 100 V; skimmer 1, 15.0 V; skimmer 2, 10.0 V). Nitrogen was used as drying gas at 150 °C. The sample solutions were introduced continuously via a syringe pump with a flow rate of 120 μl/h. All data were acquired with 512 k data points and zero filled to 2048 k by averaging 32 scans. The XMASS Software (Bruker, Version 6.1.2) was used for evaluating the data. The positive ion ESI mass spectra and the collision-induced dissociation (CID) mass spectra were obtained from a TSQ Quantum Ultra AM system equipped with a hot ESI source (HESI, electrospray voltage 3.0 kV, sheath gas: nitrogen; vaporizer temperature: 50 °C; capillary temperature: 250 °C; The MS system is coupled with a Surveyor Plus micro-HPLC (Thermo Electron), equipped with a RP18 column (5 μm, 150 × 1 mm, Hypersil GOLD, Thermo Scientific). For the HPLC a gradient system was used starting from H_2_O:CH_3_CN 90:10 (each of them containing 0.2 % HOAc) to 5:95 within 15 min and then hold on 5 % for further 30 min; flow rate 70 μl/min. Sterols were determined by GC-MS (Voyager/Trace GC 2000, Thermo Quest CE Instruments): 70 eV EI, source temp. 200 °C; column ZB-5 (Phenomenex, 30 m × 0.25 mm, 0.25 μm film thickness); inj. temp. 250 °C, interface temp. 300 °C; carrier gas He, flow rate 1.0 ml/min, constant pressure mode; splitless injection, column temp. program: 60 °C for 1 min, then raised to 300 °C at a rate of 10 °C/min to 290 °C for 15 min.

### Plant material

The plant materials were collected and identified by Elias Ndive, a botanist from Limbé Botanic Garden, on March 2009 near the town of Buea on the eastern slopes of Mount Cameroon in the South West Province of Cameroon. Voucher specimens (*H. foetidum* (L.) Moench: SCE2463, *H. mechowianum* Klatt: SCE2467) are deposited in the Herbarium of Limbé Botanic Garden.

### Extraction and isolation

Leaves and flowers of *H. foetidum* and leaves of *H. mechowianum* were extracted exhaustively with 90 % methanol for a period of 72 h. The solvent was removed by evaporation under reduced pressure. From the crude flower extract of *H. foetidum,* by purification with successive column and preparative TLC chromatography on silica gel using a chloroform/methanol gradient systems,the compounds 7,4′-dihydroxy-5-methoxy-flavanone (**1**) and kaur-16-en-18-oic acid (**6**) were obtained while the compounds 6′-methoxy-2′,4,4′-trihydroxychalcone (helichrysetin) (**2**), 6′-methoxy-2′,4-dihydroxychalcone-4′-*O*-*β*-D-glucoside (**3**), apigenin (**4**), apigenin-7-*O*-*β*-D-glucoside (**5**) were isolated from the leaves and flowers of *H. Foetidum*.

The aqueous residue of the crude extract of *H. mechowianum* leaves was partitioned successively with *n*-heptane and ethyl acetate. The *n*-heptane and the ethyl acetate extracts were further purified by silica gel column chromatography using *n*-hexane/ethyl acetate gradient systems resulting in the isolation of a phytosterol fraction and of 3,5,7-trihydroxy-8-methoxyflavone (**12**), respectively. The water fraction was further separated using Diaion HP20 eluted with water, methanol, ethyl acetate and acetone followed by chromatography of the methanol fraction on Sephadex LH20 to give 4,5-dicaffeoyl quinic acid (**13**). The crude extracts of *H. foetidum* and *H. mechowianum* were analyzed by LC-ESI-MS, MS/MS and FTICR-HRMS.

*7,4′-dihydroxy-5-methoxy-flavanone* (**1**): ^1^H NMR (DMSO-d_6_): δ 9.60 (1H, brs, OH), 7.28 (2H, d, 8.6, H2′/6′), 6.77 (2H, d, 8.6, H3′/5′), 6.04 (1H, d, 2.2, H6), 5.94 (1H, d, 2.2, H8), 5.32 (1H, dd, 12.6/2.9, H2), 3.72 (3H, s, OMe), 2.98 (1H, dd, 16.3/12.6, H3b), ca. 2.5 (1H, m, superimposed by DMSO, H3a). ^13^C NMR (DMSO-d_6_) δ 79.0(C2), 42.4(C3), 196.3(C4), 163.0(C5),104.6(C6),167.1(C7),95.4(C8),162.8(C9),101.6(C10), 129.6(C1′), 127.6(C2′/C6′), 115.0(C3′/C5′), 157.6(C4′), 55.4 (OMe).

*6′-methoxy-2′,4,4′-trihydroxychalcone* (**2**): ^1^H NMR (MeOD + CDCl_3_): δ 7.73 (1H, d, 16.0, Hα), 7.68 (1H, d, 16.0, Hβ), 7.49 (2H, d, 8.6, H2/6), 6.84 (2H, d, 8.6, H3/5), 5.99 (1H, d, 2.2, H3′),5.96 (1H, d, 2.2, H5′), 3.93 (OMe) ^13^C NMR (MeOD + CDCl_3_): δ 193.6 (C = O), 168.2 (C4′), 165.9 (C2′), 164.2 (C6′), 160.5 (C4), 143.5 (CH-β), 131.0 (C2/6), 128.0 (C1), 125.2 (CH-α), 116.6 (C3/5), 106.3 (C1′), 96.9 (C5′), 92.2 (C3′), 56.2 (OMe).

*6′-methoxy-2′,4-dihydroxychalcone-4′-O-β-D-glucoside* (**3**): ^1^H NMR (MeOD): δ 7.72 (1H, s, Hα), 7.71 (1H, s, Hβ), 7.51 (2H, d, 8.7, H2/6), 6.83 (2H, d, 8.7, H3/5), 6.31 (1H, d, 2.2, H3′), 6.24 (1H, d, 2.2, H5′), 3.95 (3H, s, OMe), glucose moiety: δ 4.99 (1H, d, 7.5, H1″), 4.24(H2″), 3.61(H3″), 3.60(H4″), 3.53(H5″), 3.80–3.90 (H6″), ^13^C NMR (MeOD) δ 193.6 (C = O), 168.2 (C4′), 165.9 (C2′), 164.2 (C6′), 160.5 (C4), 143.5 (CH-β), 131.0 (C2/6), 128.0 (C1), 125.2 (CH-α), 116.6 (C 3/5), 106.3 (C1′), 96.9 (C5′), 92.2 (C3′), 56.2 (OMe) glucose moiety: δ 73.7(C1″), 71.0(C2″), 77.2(C3″), 78.9(C4″), 79.8(C5″), 60.6(C6″),

*apigenin* (**4**): ^1^H NMR (DMSO-d_6_): δ 12.96 (1H, s, OH), 7.93 (2H, d, 8.8, H2′/6′), 6.93 (2H, d, 8.8, H3′/5′), 6.78 (1H, s, H3), 6.48 (1H, d, 2.0, H8), 6.19 (1H, d, 2.0, H6). ^13^C NMR (DMSO-d_6_): δ 180.4 (C4), 164.94 (C5), 164.4 (C2), 162.6 (C4′), 160.7 (C9), 160.1 (C7), 129.3 (C2′/ C6′), 123.1 (C1′), 117.0 (C3′/ C5′), 109.3 (C10), 106.5 (C3), 104.8 (C6), 99.3 (C8).

*apigenin-7-O-β-D-glucoside* (**5**): ^1^HNMR(CD_3_OD, 500 MHz): aglycon moiety:δ 7.83 (2H,d, 8.8,H-2′/ H-6′), 6.92 (2H,d, 8.8, H-3′/ H-5′), 6.83 (1H,d, 2.1 Hz, H-6), 6.71 (1H,d, 2.1 Hz, H-8), 6.58 (1H,s, H-3), glucose moiety: δ 4.90 (1H,d, 7.6, H-1″’), 3.50-3.25 (4H,m,H-2″, 3″, 4″, 5″), 3.87 (1H,dd, 11.9/ 2.2, H-6_b_″), 3.73 (1H,dd,11.9/ 5.4, H-6_a_″ ^13^C NMR (DMSO-d_6_): δ 182.0 (C4), 164.3 (C5), 163.0 (C2), 161.5 (C4′), 161.1 (C9), 157.0 (C7), 128.7 (C2′/C-6′), 121.0 (C1′), 116.0 (C3′/C5′), 105.4 (C10), 103.1 (C3), 99.9 (C6), 99.6 (C8), glucose moiety: δ 105.1 (C1″), 78.6 (C5″), 77.5 (C3″), 74.8 (C2″), 71.8(C4″), 62.6 (C6″)

*kaur-16-en-18-oic acid* (**6**): ^1^H NMR (CDCl_3_): δ 4.79 (1H, s, H17), 4.73 (1H, s, H17), 2.63 (1H,brs), 2.15 (1H, brd,13.9), 2.05 (2H, d, 2.0), 1.24 (3H, s, Me), 0.95 (3H, s, Me) ^13^C NMR (CDCl_3_): δ 184.1 (C = O), 155.9 (C), 103.0 (CH_2_), 57.0 (CH), 55.1 (CH) 48.9 (CH_2_), 44.2 (C), 43.8 (CH), 43.7 (C), 41.3 (CH_2_), 40.7 (CH_2_), 39.7 (CH_2_), 39.6 (C), 37.8 (CH_2_), 33.1 (CH_2_), 28.9 (CH_3_), 21.8 (CH_2_), 19.1 (CH_2_), 18.4 (CH_2_), 15.6 (CH_3_).

*3,5,7-trihydroxy-8-methoxyflavone***(12**): ^1^H NMR (CDCl_3_): δ 11.49 (1H, s, OH), 8.24 (2H, d, 7.1), 7.60-7.49 (3H, m), 6.70 (1H, s, OH), 6.46 (1H, s, OH), 6.33 (1H, s, H6), 4.05 (3H, s, OMe). ^13^C NMR (CDCl_3_): δ 175.6 (C = O), 156.6 (C), 155.4 (C), 148.0 (C), 144.9 (C), 136.5 (C), 130.7 (C), 130.4 (CH), 128.8 (2 CH), 127.5 (2 CH), 126.9 (C), 98.2 (CH), 61.9 (OMe).

*4,5-dicaffeoyl quinic acid* (**13**): ^1^H NMR (MeOD): δ 7.585/7.507 (1H, d, 16.0, H7′/7″), 7.014/6.994 (1H, d, 2.0, H2′/2″), 6.905/6.890 (1H, dd, 8.2/2.0, H6′/6″), 6.739/6.733 (1H, d, 8.2, 5′/5″), 6.273/6.190 (1H, d, 16.0, H8′/8″), 5.661 (1H, m, H5) 5.108 (1H, dd, 9.8/3, H4), 4.337 (1H, d, 3, H3), 2.284 (1H, brdd, 14.1 2.1, H2a), ca. 2.22 (2H, H6), 2.046 (1H, brd, 12.5, H2a).^13^C NMR (MeOD): δ 178.9 (C7), 168.6/168.4 (C9′/9″), 149.6 (C4′/4″), 147.6/147.4 (C7′/7″), 146.75/146.73 (C3′/3″), 127.7/127.6 (C1′/1″), 123.1 (C6′/6″), 116.4 (C5′/5″), 115.1 (C2′/2″), 114.8 (C8′/8″), 76.9 (C1), 76.6 (C4), 70.2 (C3), 69.3 (C5), 40.2 (C6), 38.7 (C2).

## Bioassays

### Protease inhibition assay

#### Procedure

Initially the extracts or purified constituents were dissolved in DMSO and the dilutions of samples tested were made in the respective buffer for each enzyme, i.e., 0.1 M sodium phosphate (pH 7.5) for subtilisin and 0.1 M sodium acetate (pH 4.4) for pepsin. Samples (0.01 - 50 μg/ml) were pre-incubated with subtilisin (37 nM) or pepsin (1.7 nM) for 30 min and then, transferred to a black opaque microplate. The substrate EDANS-DABCYL (2 μM), prepared in the specific buffer for each protease, were automatically injected. The final volume was 100 μl. Experiments were performed separately for each protease, which was prepared at the day of experiment. Reads were made for a period of 5 min, with 1 min intervals, and temperature controlled at 37 °C. The mean, standard deviation and relative standard deviation (RSD) of triplicates and the percentage of inhibition were calculated using the final fluorescence intensity measured.

#### Reagents

Pepsin from porcine gastric mucosa, Recombinant Type VIII Subtilisin Carlsberg, Arg-Glu-(EDANS)-Ser-Gln-Asn-Tyr-Pro-Ile-Val-Gln-Lys-(DALBCYL)-Arg fluorogenic substrate (EDANS-DABCYL), DMSO spectrophotometric grade were from Sigma-Aldrich, Sao Paulo, Brazil. Fluorescence bioassay data were collected with a multi detection microplate reader Synergy^TM^ HT (Bio-Tek® Instruments Inc., Winooski, Vermont, USA), with 360 nm excitation and 460 nm emission filters, and analyzed using KC4 software (Bio-Tek®Instruments) and a Microsoft Windows XP.

#### Antibacterial assay

The antibacterial activity against a fluorescent *Bacillus subtilis* [50] was determined with a fluorescence based antibacterial growth inhibition assay. The fluorescence was measured on a microtiter plate reader GENios Pro (Fa. Tecan, excitation 510 nm; emission 535 nm). The *Bacillus subtilis* strain 168 (P_AbrB_-IYFP) was maintained on TY (tryptone-yeast extract) medium supplemented with 1 % Bacto-tryptone, 0.5 % Bacto-yeast extract, 1 % NaCl and Chloramphenicol (5 μg/ml). Erythromycin was used as positive control for growth inhibition.

#### Antifungal assay

The antifungal activity against the phytopathogenic fungus *Cladosporium cucumerinum* was tested by bioautography on silica gel plates [51] in concentrations of 50, 100, 200 and 400 μg/cm. Amphotericine B was used as positive control for growth inhibition.

#### Cytotoxicity assay

The cytotoxicity was determined by XTT method, using the Cell Proliferation Kit II (Roche). The human prostate cancer cell line PC-3 was maintained in RPMI 1640 medium supplemented with 10 % fetal bovine serum, 1 % L-alanyl-L-glutamin (200 mM), 1 % penicillin/streptomycin and 1,6 % hepes (1 M). For the measurement of cytotoxicity the same medium was used without antibiotics. For PC-3 500 cells/well were seeded overnight into 96-well plates and exposed to serial dilution of each compound for three days.

#### Molecular modeling

All molecular modelling was carried out on a Linux workstation running on a 3.5 GHz Intel Core2 Duo processor (Santa Clara, USA). Low energy 3D structures of the thirteen isolated compounds were generated using the MOE software package [52] and the Merck molecular forecefiled [53] and saved in mol2 format. These were initially treated with LigPrep [54], distributed by Schrodinger, Inc (Camberley, UK). This implementation was carried out with the graphical user interface (GUI) of the Maestro software package (New York, USA) [55], using the OPLS force field [56–58]. Protonation states at biologically relevant pH were correctly assigned (group I metals in simple salts were disconnected, strong acids were deprotonated and strong bases protonated, while topological duplicates and explicit hydrogens were added). A set of the ADMET-related properties (a total of 46 molecular descriptors) were calculated using the QikProp program (New York, USA) [46] running in normal mode. QikProp generates physically relevant descriptors and uses them to perform ADMET predictions. An overall ADME-compliance score, drug-likeness parameter (indicated by #stars), was used to assess the pharmacokinetic profiles of the compounds. The #stars parameter indicates the number of property descriptors computed by QikProp, which fall outside the optimum range of values for 95 % of known drugs. The methods implemented were developed by Jorgensen *et al.* [47–49].

## Conclusion

Eight known compounds have been identified from the extracts of two species from the genus *Helichrysum* (Compositae) harvested from the South West of Cameroon (Central Africa). The results showed that the flavonoid glycosides (**3, 5**) from *H. foetidum* exhibited protease inhibition, while the compound (**13**) from *H. mechowianum* contribute to the stomach protecting effects. In addition, the antibacterial and antifungal activities of compound (**6**) was demonstrated by the fact that it was found to possess a potent inhibitor effect against the tested microorganisms. The differential bioactivities and determined constituents support the traditional use of the species. Molecular modelling studies showed that five of the isolated compounds showed physicochemical properties that completely within the recommended range for more that 95 % of known drugs, while two compounds have only one violation.

## Additional file

Additional file 1: Figure S1. LC-MS of Helichrysum mechowianum, MeOH fraction positive and negative ion. **Figure S2**. LC-MS of Helichrysum foetidum leaf extract positive and negative ion.
