# Supplementary material for: Protease-inhibiting, molecular modeling and antimicrobial activities of extracts and constituents from Helichrysum foetidum and Helichrysum mechowianum (compositae)
Source: Chem Cent J. 2015 May 30;9:32. doi: 10.1186/s13065-015-0108-1 (PMC4452611; doi:10.1186/s13065-015-0108-1)
Supplement: Additional file 1: Figure S1. — LC-MS of Helichrysum mechowianum, MeOH fraction positive and negative ion. Figure S2. LC-MS of Helichrysum foetidum leaf extract positive and negative ion. [file 13065_2015_108_MOESM1_ESM.docx]

**Protease-inhibiting and antimicrobial activities of extracts and constituents from *Helichrysum foetidum* and *Helichrysum mechowianum* (Compositae.**

**Fanny-Aimée Essombe Malolo ^a^, Achille Bissoue Nouga ^b^,** **Antoine Kakam ^b^,**  **Katrin Franke^d^, Lidwine Ngah ^a^, Otavio Jerome Flausino ^c^, Emmanuel Mpondo Mpondo^a^, Fidele Ntie-Kang^d^*, Jean Claude Ndom ^a,^*, Vanderlan da Silva Bolzani^c^ Ludger Wessjohann^e^***

*^a^Department of Pharmacy, University of Douala, Douala, Cameroon P.O. Box 812*

*^b^Department of Chemistry, University of Douala, Douala, Cameroon P.O. Box 24157*

*^c^Química, Departamento de Química Orgânica, Núcleo de Bioensaio, Biossíntese e Ecofisiologia de Produtos Naturais – NuBBE,– Universidade Estadual Paulista (UNESP), Araraquara – São Paulo, Brazil Instituto de Rua Prof. Francisco Degni s/n – 14.800-900*

*^d^Department of Bioorganic Chemistry, Leibniz Institute of Plant Biochemistry, Weinberg 3, D-06120 Halle (Saale), Germany*

*^e^Chemical and Bioactivity Information Centre, Department of Chemistry, University of Buea, P. O. Box 63, Buea, Cameroon*

F.A.E.M.: emfay1@yahoo.fr

A.B.N.: anbissoue@yahoo.fr

A.K .: [amkakam@yahoo.fr](mailto:amkakam@yahoo.fr)

K. Franke.: Katrin. Franke@ipb-halle.de

L.N.: lidwingah@yahoo.fr

O.J.F.: *Flausino*@usp.br

E.M.M. : tonjoli@yahoo.fr

F.N.K. : ntiekfidele@gmail.com

J.C.N. ndomjefr@yahoo.fr

V.S.B.: bolzani@gmail.com

L. A. W.: wessjohann@ipb-halle.de

^*^Corresponding authors:  *Associate Prof*. *Jean Claude Ndom. University of Douala, PO. Box 24157, Cameroon, Tel: +237 76600549; E-mail: ndomjefr@yahoo*.*fr* ; Dr. Fidele Ntie-Kang, *Chemical and Bioactivity Information Centre, Department of Chemistry, University of Buea, P. O. Box 63, Buea, Cameroon; E-mail:ntiekfidele@gmail.com, Tel.: +237 77915473, E-mail:* [*ntiekfidele@gmail.com*](mailto:ntiekfidele@gmail.com)*; Prof. Dr. Ludger A. Wessjohann, Leibniz Institute of Plant Biochemistry, Weinberg 3, D-06120 Halle (Saale), Germany. Tel. +49 (345) 5582-1301, Fax +49 (345) 5582-1309, E-mail: wessjohann@ipb-halle,de.*

SUPPLEMENTARY DATA
